# Whole Exome Sequencing Study Identifies Novel Rare Risk Variants for Habitual Coffee Consumption Involved in Olfactory Receptor and Hyperphagia

**DOI:** 10.3390/nu14204330

**Published:** 2022-10-16

**Authors:** Bolun Cheng, Chuyu Pan, Shiqiang Cheng, Peilin Meng, Li Liu, Wenming Wei, Xuena Yang, Yumeng Jia, Yan Wen, Feng Zhang

**Affiliations:** Key Laboratory of Trace Elements and Endemic Diseases, Collaborative Innovation Center of Endemic Disease and Health Promotion for Silk Road Region, School of Public Health, Health Science Center, Xi’an Jiaotong University, Xi’an 710061, China

**Keywords:** coffee consumption, exome-wide association study, olfactory receptor, hyperphagia, nervous system

## Abstract

Habitual coffee consumption is an addictive behavior with unknown genetic variations and has raised public health issues about its potential health-related outcomes. We performed exome-wide association studies to identify rare risk variants contributing to habitual coffee consumption utilizing the newly released UK Biobank exome dataset (*n* = 200,643). A total of 34,761 qualifying variants were imported into SKAT to conduct gene-based burden and robust tests with minor allele frequency <0.01, adjusting the polygenic risk scores (PRS) of coffee intake to exclude the effect of common coffee-related polygenic risk. The gene-based burden and robust test of the exonic variants found seven exome-wide significant associations, such as *OR2G2* (*P_SKAT_* = 1.88 × 10^−9^, *P_SKAT-Robust_* = 2.91 × 10^−17^), *VEZT1* (*P_SKAT_* = 3.72 × 10^−7^, *P_SKAT-Robust_* = 1.41 × 10^−7^), and *IRGC* (*P_SKAT_* = 2.92 × 10^−5^, *P_SKAT-Robust_* = 1.07 × 10^−7^). These candidate genes were verified in the GWAS summary data of coffee intake, such as rs12737801 (*p* = 0.002) in *OR2G2*, and rs34439296 (*p* = 0.008) in *IRGC*. This study could help to extend genetic insights into the pathogenesis of coffee addiction, and may point to molecular mechanisms underlying health effects of habitual coffee consumption.

## 1. Introduction

Coffee is among the most popular drinks in the world [1]. North American coffee drinkers usually consume two cups per day, whereas European drinkers consume at least four cups per day [2]. In prospective cohort studies, habitual coffee consumption has been related to a decreased risk of Alzheimer’s disease, Parkinson’s disease [3], and type II diabetes [4]. However, the influences of coffee consumption on some cancers [5], cardiovascular disease [6], myocardial infarction [7], and other health-related phenotypes remain controversial. For most people, coffee is the main source of caffeine and a form of addictive behavior [1,8]. Caffeine withdrawal has been listed in the Diagnostic and Statistical Manual of Mental Disorders-5 (DSM-5), and the severity of caffeine use disorder is affected by the amount of caffeine consumption [9]. Habitual coffee consumption has raised public health issues of its potential health-related outcomes [10]. Knowledge of factors contributing to habitual coffee consumption may facilitate public knowledge and clinical research on coffee intake.

Coffee consumption varies greatly between individuals within populations, which was shown to be affected by coffee preferences and genetic variation [11]. Genetic studies in twins indicated that the heritability of coffee and caffeine intake was estimated to be between 0.39 and 0.56 [12,13]. The quantile-specific heritability regression showed that there were differences in estimated heritability between heavier and lighter coffee intake [14]. Most genetic studies of coffee intake have focused on caffeine and have limited the candidate gene to polymorphisms in *ADORA2A* and *CYP1A2* [15,16]. Genome-wide association studies (GWAS) of habitual caffeine and coffee consumption identified coffee-related variants closed to *CYP1A2* and *AHR* [17,18]. Later, the Coffee and Caffeine Genetics Consortium conducted a meta-GWAS of habitual coffee consumption, and identified six novel loci located in or close to genes potentially related to pharmacokinetics (*ABCG2*, *AHR*, *POR*, and *CYP1A2*) and pharmacodynamics (*BDNF* and *SLC6A4*) [2]. Recently, epigenome-wide association studies for coffee consumption revealed 11 candidate CpGs annotated to the *AHRR*, *HDAC4*, *GFI1*, *F2RL3*, *FLJ43663*, and *PHGDH* genes [19]. Thus far, few rare variants of habitual coffee consumption have been identified, and most of its genetic architecture remains unknown.

To date, GWASs have identified many variants at genomic loci related to polygenetic traits in a large cohort of population, particularly between common single-nucleotide polymorphisms (SNPs) and complex diseases [20]. However, linkage disequilibrium (LD) and population stratification make it convoluted for GWAS to identify exact causal variants [20]. In addition, most of the genetic variants investigated by GWASs are common in the population, due to their minor allele frequency (MAF) typically being greater than 1% [21]. Although these common DNA sequence variations produce many reproducible associations for GWAS in complex traits, it remains challenging to understand the biological underpinnings of complex phenotypes [22]. Exome-wide association studies (exome-WAS) found that rare coding variants (MAF <1%) were likely to have stronger phenotypic effects than common variants and jointly contribute to the heritability of complex traits [23,24]. Recently, by using 10,900 whole-exome sequences linked to electronic health records data, Park et al. identified new gene–phenotype associations of the cumulative effects of rare coding variants on human disease [25]. However, identifying pathogenic genes and rare coding variants of habitual coffee consumption remains a huge challenge.

It has been reported that rare risk variants are more widespread among patients with low polygenic risk scores (PRS) of disease [26]. Lu et al. indicated that individuals with low polygenic risk for disease may be more likely to harbor rare variants than people with high polygenic risk [26]. Identifying rare risk variants for disease may be important for clinical care via adjusting diagnostic and treatment strategies [27]. Recently, we identified novel risk rare variants associated with depression in participants with low PRS of depression via exome-WAS [28]. In this study, we aimed to identify rare pathogenic variants for the phenotype of habitual coffee consumption but exclude the effect of common polygenic risk associated with coffee intake. We generated PRS for coffee intake and identified rare pathogenic variants among 20,566 individuals with the exome sequencing dataset from UK Biobank by adjusting the levels of polygenic risk.

## 2. Materials and Methods

### 2.1. Ethic Statement

Ethical approval of UK Biobank was granted by the National Health Service National Research Ethics Service (reference 11/NW/0382).

### 2.2. Study Participants from UK Biobank

Our study samples were driven from the UK Biobank (UKB), a widely used resource for genetic research due to its large-scale database and wealth of genetic and health information [29]. The UKB recruited about half a million participants aged between 40 and 69, and collected a large amount of phenotypic and health information for everyone, including physical and biological measurements, lifestyle indicators, and genome-wide genotyping from 2006 to 2010 [29]. The UK Biobank resource with application No. 46478 was used in this study. Health-related records for each participant were obtained from screenshot questions or verbal interviews within the assessment center, including age, sex, alcohol use frequency/week, smoking frequency/day, energy, body mass index (BMI), and Townsend deprivation index (TDI).

### 2.3. UK Biobank Genotyping and Imputation for PRS Calculation

The UK Biobank performed high-quality genome-wide genotyping and genotype imputation [29]. Briefly, genome-wide genotyping for DNA samples was conducted using either the Affymetrix UK BiLEVE Axiom (807,411 markers) or Affymetrix UK Biobank Axiom (825,927 markers) array. Imputation for SNPs was performed by IMPUTE2 against the reference panel of the Haplotype Reference Consortium, 1000 Genomes, and UK10K projects [29]. Detailed information of the array design, genotyping, and quality control procedures are available elsewhere [29].

### 2.4. Exome Sequencing, Genotype Calling, and Data Processing in UK Biobank

The exome data were obtained from the UK Biobank exome releases, including 200,643 participants who participated in exome sequencing and genotype calling via the UK Biobank Exome Sequencing Consortium (UKB-ESC). We used the hg38 assembly with coverage exceeding 20X at 95.6% of the sites on average [30]. The IDT xGen Exome Research Panel v1.0 was used to capture the exomes. BWA-MEM was used in the OQFE protocol to map raw reads (Fastq) to the hg38 reference in a deterministic manner, preserving all supplementary alignments [30]. DeepVariant 0.10.0 was used to call variants on each CRAM via a deep learning model retrained on exome data. The genomic Variant Call Format (gVCF) was generated for each sample containing all variant genotypes by limiting variant calls to the exome capture region and the 100 base-pairs flanking each capture target. GLnexus 1.2.6 was used for merge and joint-genotyping of all gVCFs via the default ‘DeepVariantWES’ parameters to build a single multi-sample VCF (pVCF) for all 200,643 UKB samples [30]. PLINK format files were directly derived from this pVCF. The OQFE version of the UKB exome files (Data-field 23155, PLINK format-interim 200 k release) were utilized in our subsequent analysis.

### 2.5. Habitual Coffee Consumption Definition

The phenotype of habitual coffee consumption was defined based on the coffee intake from the diet category of UK Biobank (Data-Field 1498). The units of measurement for coffee intake were in number of cups per day (cups/day). The averages of coffee intake over the last year for all participants were collected by the UKB Assessment Centre via the touchscreen question, “How many cups of coffee do you drink each day? (Include decaffeinated coffee)”. Answers less than 0 or greater than 99 were rejected, and answers greater than 10 were asked for confirmation. The numbers of coffee consumption cups were mean-centered and normalized to one standard deviation (SD) before subsequent analysis. The participants were restricted to only “White British”, based on ancestry. The participants who reported discordance between self-reported sex and genetic sex, were genotyped but not imputed, and who did not attend exome sequencing were excluded in this study. Finally, 20,566 participants of habitual coffee consumption were included.

### 2.6. Filtering and Annotation of Genetic Variants

The single nucleotide variations (SNVs) with MAF >0.01, missing call rates <0.1 were excluded from annotation among all variants and samples. ANNOVAR was used to annotate all variants on human genome hg38 [31]. In population-based genetic analysis, the most common strategy for grouping rare variants together is at the gene level, often through a gene-based collapsing test [32]. For exome sequencing data, genes are the natural units for collapsing genetic variants, and collapsing rare variants may help to identify disease-associated molecular mechanisms by enriching the genetic signal [33]. In rare variant association tests, the hypothesis of burden tests is that all rare variants in the target region have influences on the phenotype in the same direction and of similar magnitude [34]. Burden tests can have stronger power if a high proportion of the rare variants in a region are truly causal and affect the phenotype in the same direction [35]. All non-benign coding variants including the frameshift variant, non-frameshift variant, non-synonymous, start-loss, stop-loss, and stop-gain were included in the gene-based burden test [28].

### 2.7. Polygenic Risk Scores Calculation for Habitual Coffee Consumption

A total of 14 SNPs were first obtained from a recent large GWAS of habitual coffee consumption, which consisted of approximately half a million participants of European ancestry from UK Biobank [36]. The variants with a kinship coefficient > 0.0442, minor allele frequency < 0.001, or low imputation quality score ≤ 0.3 were removed based on heterozygosity and missingness. Details of genotyping, imputation, quality control, and statistical analysis were available in the published study [36]. Briefly, we used PRsice-2 software to calculate PRS for habitual coffee consumption [37]. Based on a previous study, we set the clustering algorithm to identify SNPs within 500kb in LD with an r2 > 0.2, and used age, sex, and 1–10 principle components of population structure (PC) as covariates. The best model was derived by testing SNP inclusion with a range of *p* values (5 × 10^−8^ to 1 interval of 0.05) in the dataset, to test which threshold gave the maximum Nagelkerke R2 value for each participant to generate PRS. We determined that the *p* value threshold of 5 × 10^−8^ for habitual coffee consumption (Nagelkerke R^2^max = 0.34%) was the optimal inclusion threshold for in this study.

### 2.8. Gene-Based Association Analyses

The exome data of binary PLINK format were loaded into SKAT R-package to conduct a gene-based burden test, examining the aggregate effect of variants within a region defined by gene annotations [38]. The gene-based burden and robust test were performed by using the “SKATBinary.SSD.All” and the “SKATBinary_Robust.SSD.All” function, respectively [39,40]. According to the previous study, the minimum parameter of aggregated alleles for a gene-based test was set at 10 [41]. Only genes with at least two eligible variants were included in the analysis. The strict Bonferroni correction was performed for multiple testing, according to the total number of tests done for the genes. Age, sex, smoking history, alcohol intake, energy, BMI, TDI, 1–10 PCs, and coffee intake PRS were used as covariates in our SKAT analysis. The strict Bonferroni adjusted *p* value was used to define statistical significance (*P_Bonf_* < 5 × 10^−5^).

### 2.9. Verification for Gene-Based Association Analyses Results

A large-scale GWAS summary data of coffee intake (ukb-b-5237) from the MRC-IEU consortium was used to validate the reliability of an exome-wide association study of habitual coffee consumption (accessed on 15 June 2022). Briefly, a GWAS was performed based on 428,860 individuals with European ancestry. Detailed descriptions of this data set are available here (https://gwas.mrcieu.ac.uk/datasets/ukb-b-5237/ accessed on 17 September 2022).

## 3. Results

### 3.1. Population Characteristic of Habitual Coffee Consumption

In this study, a total of 20,566 participants of habitual coffee consumption (mean age, 56.55 years; 45% male) were included after quality control. The detailed basic characteristics of habitual coffee consumption individuals recruited in this study are presented in Table 1.

### 3.2. Annotation of Identified Variants

After excluding SNVs with MAF > 0.01 and missing call rates < 0.1, a total of 64,901 variants were annotated, including 335 non-frameshift variant, 148 frameshift variant, 33,758 non-synonymous, 29,772 synonymous, 393 stop-gain, 91 start-loss, 36 stop-loss, and 368 unknown variants. We removed the benign coding variants, including 29,772 synonymous and 368 unknown variants, leaving 34,761 eligible variants in the subsequent gene-based burden test.

### 3.3. Gene-Based Burden Test Result

**Figure 1:**
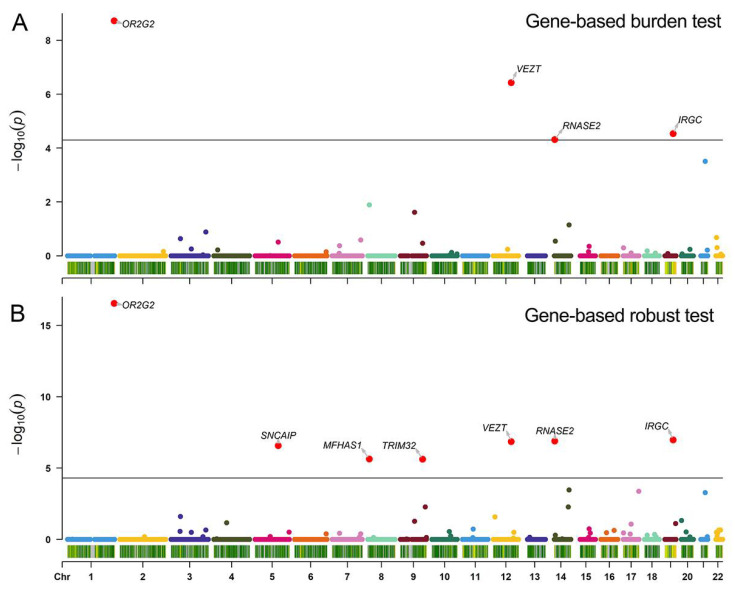
Manhattan plots of the exome-wide association results of rare habitual coffee consumption-related variants. (**A**) The exome-wide association results for gene-based burden tests. (**B**) The exome-wide association results for gene-based robust tests. The plots show the gene-based Bonferroni adjusted *p* values against their genomic position for association with habitual coffee consumption. Each dot on the x-axis represents a gene, and the association strength on the y-axis represents the −log10 (Bonferroni adjusted *p* value) from SKAT burden test or SKAT robust burden test aggregating rare variants (MAF < 0.01) by gene. Chr, chromosome.

A total of 12,122 genes with at least two variants were included in gene-based analysis. The gene-based burden and robust test of the exonic variants detected 4 and 7 exome-wide significant associations, respectively. For example, *OR2G2* (*P_SKAT Bonferroni adjust_* = 1.88 × 10^−9^, *P_SKAT Robust Bonferroni adjust_* = 2.91 × 10^−17^), *VEZT1* (*P_SKAT Bonferroni adjust_* = 3.72 × 10^−7^, *P_SKAT Robust Bonferroni adjust_* = 1.41 × 10^−7^), *IRGC* (*P_SKAT Bonferroni adjust_* = 2.92 × 10^−5^, *P_SKAT Robust Bonferroni adjust_* = 1.07 × 10^−7^), and *RNASE2* (*P_SKAT Bonferroni adjust_* = 4.85 × 10^−5^, *P_SKAT Robust Bonferroni adjust_* = 1.29 × 10^−7^) were associated with habitual coffee consumption. Detailed information about the significant genes are presented in Figure 1 and Table 2. The detailed results of gene-based burden and robust test are summarized in Tables S1 and S2.

### 3.4. Verification for Gene-Based Association Analyses Results

The genes identified in the gene-based association analyses for habitual coffee consumption were verified in a coffee intake cohort from the MRC-IEU consortium. We observed six candidate SNPs corresponding to *OR2G2*, *VEZT*, *IRGC*, and *SNCAIP* in the GWAS datasets (*p* < 0.05). For example, rs12737801 (*p* = 0.002) and rs1151687 (*p* = 0.002) were verified within the *OR2G2* region, and rs34439296 (*p* = 0.008) and rs346049 (*p* = 0.011) were verified within the *IRGC* region (Table 3).

## 4. Discussion

In this study, our aim was to detect rare pathogenic variants in individuals with habitual coffee consumption to broaden our knowledge and understanding of the genetic characteristics of coffee addiction. We performed a gene-based exome-wide association study by using a large-scale exome dataset from the UK Biobank, and detected seven novel candidate genes for habitual coffee consumption, after excluding the effect of coffee-related common polygenic risk.

Our results highlight a vital role of the olfactory receptor underlying a genetic propensity to coffee consumption. *OR2G2* (Olfactory receptor family 2 subfamily G member 2), also known as *OR1-32*, is related to olfactory receptor activity. Olfactory receptors interact with odorant molecules in the human nose to initiate neuronal responses that trigger the sense of smell [42]. Olfactory receptors span a seven-transmembrane domain, with many neurotransmitters responsible for the recognition of odorant signals [43]. Malnic et al. determined the chromosomal position of each intact olfactory receptor gene and olfactory receptor pseudogene, and identified *OR2G2* loci distributed in the 1q44 chromosome [44]. Chromosome 1q44 is the key genetic region related to a variety of neurological disorders in human [45]. Deletions in the 1q44 region have been confirmed in several abnormal developments of the brain, including agenesis of the corpus callosum (ACC) [46] and seizures [47]. *OR2G2* was identified as a candidate gene for habitual coffee consumption in our study, which has been confirmed to be associated with olfactory receptors by previous studies. Future investigation is needed to explore its potential molecular mechanisms in habitual coffee consumption.

*SNCAIP* (Synphilin-1) encodes a protein interacting with alpha-synuclein in neuronal tissue, which may influence the onset of neurodegeneration [48]. Shishido et al. suggested that *SNCAIP* might play a neuroprotective role in dopaminergic cells by maintaining mitochondrial function and restraining early steps in the apoptotic pathway [49]. Recent studies have indicated that *SNCAIP* is involved in hyperphagia in animal models. For example, the AMPK signaling pathway in Drosophila neurons played a vital role in *SNCAIP*-induced hyperphagia [50]. Li et al. characterized a human *SNCAIP* transgenic mouse by assessing *SNCAIP* expression, food ingestion, and spontaneous activity to confirm main behavioral changes and outcomes. They identified a novel feature of *SNCAIP* in controlling food intake [51]. Although these findings are encouraging for ongoing efforts, they also highlight the need for future studies to explore pathways of genes.

*TRIM32* variants have been associated with olfactory performance and neurodevelopment. In the mouse brain, *TRIM32* knockout provided sufficient support for neurogenesis damage caused by the loss of the cell fate determinant. *TRIM32* has been shown to cause declines in olfactory performance and deregulation of metabolomic pathways associated with mood disorders [52]. In the adult mammalian brain, neural stem cells in the subventricular region constantly produce new neurons for the olfactory bulb (OB) [53]. Hillje et al. demonstrated that *TRIM32* was necessary to induce differentiation of adult neural stem cells into OB neurons, and highlighted the role of the cell fate-determinant *TRIM32* for balanced activity during neurogenesis [53]. Genome-wide studies have found rare copy number variants (CNVs) interfering with the *TRIM32* gene at the 9q33.1 locus in some individuals with neurodevelopmental disorders. Common phenotypes observed in these individuals included autism spectrum disorder, anxiety, speech delay, attention-deficit/hyperactivity disorder, and obsessive compulsive disorder [54]. Previous studies found that regulation of *TRIM32* was also related to the recovery of neurological function [55], migration and differentiation of Schwann cells [56], synaptic downscaling [57], declined concentration of neurofilaments, and a reduction in myelinated motor axon diameters [58]. Based on the above research, *TRIM32* identified in our study was functionally associated with neurodevelopmental traits. Further mechanism studies are needed to investigate their underlying roles in the development of habitual coffee consumption.

Using the GWAS summary data of coffee intake, we verified association signals of several SNPs in the region of the target genes. The power for GWAS testing for individual rare variants may usually be low [59], resulting in weak association signals in GWAS data for SNPs corresponding to genes detected in gene-based exome-WAS. Our study is a vital process in understanding the genetics of habitual coffee consumption. Genome exome sequencing technology facilitates the discovery of low-frequency rare variants of complex traits. Besides, using PRS to correct the influence of common risk loci for coffee intake could identify coffee-related rare variants without reducing the sample size. Caffeine is a widely used psychoactive drug worldwide, and coffee is the most common form of caffeine consumption. Thus, our findings have implications for understanding individual differences in habitual coffee consumption. Other studies have investigated interactions among long-term coffee consumption, common variants, and disease risk, and our results could inform future research in these areas. There are three limitations in our study. First, the participants with coffee intake phenotypes in this study did not define how many participants used decaffeinated coffee. Our results may not be applicable for caffeine-related studies. Second, the participants who consumed coffee, but not on a daily basis, were not distinguished in the touchscreen question of coffee intake. Third, both exome sequencing samples and PRS data samples were from UKB; this potential overlap of samples may slightly affect the results of the analysis.

Caffeine-seeking behavior may explain the continual consumption of coffee despite its bitter taste [60]. Besides, the taste of coffee can be manipulated by the addition of sweetener and milk. Our recent study confirmed the potential effect of bitter or sweet beverage perception on brain function and identified several coffee-related genes [61]. Ong et al. also revealed bitter perception was causally related to intake of coffee, suggesting a role of bitter taste in the development of bitter beverage consumption [62]. Marilyn C et al. found that caffeine sensitivity was more strongly correlated with coffee taste preference than with bitter perception, and the interaction of taste preferences and physiological effects of caffeine may reflect conditioned taste preferences [63]. Thus, coffee consumption depends on the perception of taste, as well as environmental and genetic factors. Further exploration of coffee taste preference would be important for understanding coffee consumption behavior.

## 5. Conclusions

Overall, we calculated PRS for habitual coffee consumption and identified novel rare pathogenic variations in habitual coffee consumption using whole exome sequencing data from UK Biobank. The genes identified in this study were associated with the olfactory receptor, hyperphagia, and the nervous system. The results of this study may help understand the biological mechanism of rare mutations in the development of habitual coffee consumption and provide novel insights into the etiology of coffee addiction.

## Figures and Tables

**Table 1:** Basic characteristics of study participants.

*n* = 20,566	Mean ± SD	Range
Age, years	56.55 ± 7.94	40–70
Coffee intake, cups/day	2.14 ± 2.10	0–10
Coffee intake, PRS	0.01 ± 0.01	−0.02–0.04
Smoking, frequency/day	5.47 ± 9.64	0–80
Alcohol use, frequency/week	8.76 ± 9.44	0–235
Energy	8861.30 ± 3066.13	1009.84–41,830.10
BMI	27.04 ± 4.56	15.2–63.4
TDI	−1.62 ± 2.68	−6.26–9.64

Notes: Coffee intake 0 cups/day indicates no coffee intake over the last year. Energy indicates the estimated total energy intake, based on food and beverage consumption yesterday, excluding any supplements. PRS, polygenic risk score; BMI, body mass index; TDI, Townsend deprivation index.

**Table 2:** Gene-based association analysis results of habitual coffee consumption.

Gene	No. of Marker Test	*P_SKAT Bonferroni adjust_*	*P_SKAT Robust Bonferroni adjust_*
*OR2G2*	5	1.88 × 10^−9^	2.91 × 10^−17^
*VEZT*	3	3.72 × 10^−7^	1.41 × 10^−7^
*IRGC*	6	2.92 × 10^−5^	1.07 × 10^−7^
*RNASE2*	2	4.85 × 10^−5^	1.29 × 10^−7^
*SNCAIP*	6	/	2.72 × 10^−7^
*MFHAS1*	2	/	2.32 × 10^−6^
*TRIM32*	5	/	2.42 × 10^−6^

**Table 3:** Verification of gene-based association analyses results in gene region.

SNP	Gene	Chromosome	REF	ALT	GWAS P
rs12737801	*OR2G2*	1	C	G	0.002
rs1151687	*OR2G2*	1	G	C	0.002
rs201317857	*VEZT*	12	C	A	0.020
rs34439296	*IRGC*	19	C	T	0.008
rs346049	*IRGC*	19	C	T	0.011
rs55712196	*SNCAIP*	5	G	C	0.028

## Data Availability

The UK Biobank data are available through the UK Biobank Access Management System at https://www.ukbiobank.ac.uk/ (accessed on 25 May 2022). We will return the derived data fields following UK Biobank policy; in due course, they will be available through the UK Biobank Access Management System.

## Supplementary Materials

The following supporting information can be downloaded at: https://www.mdpi.com/article/10.3390/nu14204330/s1, Table S1: The results of gene-based burden test by using SKAT R-package; Table S2: The results of gene-based robust test by using SKAT R-package.
